# Phenotypic, molecular detection and antibiogram analysis of *Aeromonas Hydrophila* from *Oreochromis Niloticus* (Nile Tilapia) and Ready-To- eat fish products in selected Rift Valley lakes of Ethiopia

**DOI:** 10.1186/s12917-023-03684-3

**Published:** 2023-08-12

**Authors:** Nebiyu Kassa Kerigano, Tesfaye Rufael Chibsa, Yitbarek Getachew Molla, Abde Aliy Mohammed, Mekdes Tamiru, Abebe Olani Bulto, Tafesse Koran Wodaj, Dereje Shegu Gebreweld, Alemu Kebede Abdi

**Affiliations:** 1 Department of Fish Disease Research and Diagnostics, Animal Health Institute, Sebeta, Ethiopia; 2 Animal Health Institute, Sebeta, Ethiopia; 3 College of Veterinary Medicine and Agriculture Department of Clinical Studies, Addis Ababa University, Debrezeit, Ethiopia; 4 Department of Molecular Biology, Animal Health Institute, Sebeta, Ethiopia; 5 Department of General Bacteriology, Animal Health Institute, Sebeta, Ethiopia

**Keywords:** *A. hydrophila*, Ethiopia, Nile tilapia, qPCR, Rift valley lake

## Abstract

**Background:**

*Aeromonas hydrophila* is a zoonotic bacterial pathogen that frequently causes disease and mass mortalities among cultured and feral fishes worldwide. In Ethiopia, *A. hydrophila* outbreak was reported in Sebeta fish ponds and in Lake Tana fishery. However, there is no to little information on the molecular, and phenotypical characteristics of *A. hydrophila* in Ethiopian fisheries. Therefore, a cross-sectional study was conducted from November 2020 to May 2021 in selected Ethiopian Rift valley lakes.

**Results:**

A total of 140 samples were collected aseptically from fish (Muscle, Gill, Intestine, Spleen and Kidney) from fish landing sites, market and restaurants with purposive sampling methods. Aeromonas selective media (AMB), morphological and biochemical tests were used to isolate and identify *A. hydrophila*. Accordingly, the pathogen was isolated from 81 (60.45%) of samples. Among the isolates 92.59% expressed virulence trait through β hemolysis on blood agar media with 5% sheep blood. Moreover, 54 strains (66.67%) were further confirmed with Real-Time PCR (qPCR) using ahaI gene specific primers and optimized protocol. The highest (68.51%) were detected from live fish, (24.07%) were from market fish and the lowest (7.4%%) were from ready-to-eat products. Antibiogram analysis was conducted on ten representative isolates. Accordingly, *A. hydrophila* isolates were susceptible to ciprofloxacin (100%), chloramphenicol (100%) and ceftriaxone (100%). However, all ten isolates were resistant to Amoxicillin and Penicillin.

**Conclusions:**

The study indicates *A. hydrophila* strains carrying virulence ahaI gene that were ß-hemolytic and resistant to antibiotics commonly used in human and veterinary medicine are circulating in the fishery. The detection of the pathogen in 140 of the sampled fish population is alarming for potential outbreaks and zoonosis. Therefore, further molecular epidemiology of the disease should be studied to establish potential inter host transmission and antibiotic resistance traits. Therefore, raising the public awareness on risk associated with consuming undercooked or raw fish meat is pertinent.

## Background

Nile tilapia (*Oreochromis niloticus*) is one of the commercially important fast-growing and well adapted freshwater fish that is produced extensively and intensively all over the world [1]. Tilapia are increasingly used in aquaculture and is currently the second most important freshwater fish farmed worldwide with an annual global production of 6.4 MT [2]. It is characterized by their reasonable resistance to diseases and its suitability for intensive farming which subsequently leads to increased production and makes it as a cheap protein source for all people [3]. Nowadays this high protein source is threatened by bacterial diseases especially those caused by drug resistance and highly virulent bacteria such as *A. hydrophila* [4]. Significantly impeding both economic and socioeconomic developments in regions dependent on aquaculture and fisheries and zoonotic implications as well [5].

*A. hydrophila* is facultative anaerobic, Gram-negative bacteria that belong to the family Aeromonadaceae which is cosmopolitan in distribution and have a broad host spectrum with both cold and warm blooded animals including humans [6]. *A. hydrophila* is a wellknown bacterial pathogen that frequently causes disease and mass mortalities among cultured and feral fishes worldwide [7]. *A. hydrophila* has gained increased attention due to pathogenicity to humans and emerged as a foodborne pathogen of extreme importance [8]. *A. hydrophila* resulting serious health condition and death associated with consumption of frozen fish in market-sold sushi products containing raw fish [9]. High antibiotic resistance is seen in *A. hydrophila* infections [10] and regarded universally exhibit resistance to the penicillin for quite a long time [11] nowadays, becoming a serious public health concern. In Ethiopia however, less attention has been given to pathogens of fish including those which have zoonotic importance except few isolated cases [12]. For instance, a survey of bacterial and parasitic fish pathogens was conducted in Lake Ziway but *A. hydrophila* was not included [13]. *A. hydrophila* was reported as the most frequent isolate from Lake Tana and also the pathogen was associated with outbreak and mortality in Sebeta fish ponds [14].

In Ethiopia, intensive and semi-intensive aquaculture is becoming an emerging business in the country. The number of private investors interested in fish farming in the country is evolving and some of them have even already started the process. The Great Renaissance Dam and several other dams and reservoirs are being constructed in the country for hydropower generation, irrigation and other purposes apart from providing water for their primary uses, these water bodies could also be stocked with different fish species which could provide a source of livelihood to many rural young Ethiopians engaged in fishing. Despite the potential contribution of fisheries in the country emerging zoonotic bacterial pathogen like *A. hydrophila* could constrain the productivity and safety of the fish industry in the country. This calls for proactive investigation into important pathogens in water bodies with high fish sources in Rift Valley lakes of Ethiopia.

According to FAO [1], majority of fish catch in Ethiopia originate from Rift valley lakes. Therefore, knowing the infection status and characteristics of *A. hydrophila* in fish and ready-to-eat fish products is paramount to the understanding of the epidemiology and associated risks to public health. To this end the present study was intended to isolate and determine phenotypic and genotypic features of *A. hydrophila* infecting tilapia in selected Rift Valley Lakes and fish products in respective towns. The specific objectives of the study were to isolate *A. hydrophila* from fish and ready-to-eat fish products, to determine the susceptibility of *A. hydrophila* isolates to major antimicrobials of veterinary and human importance and to reveal phenotypic and genotypic traits of *A. hydrophila* isolates.

## Results

### Clinical and post-mortem findings

**Fig. 1:**
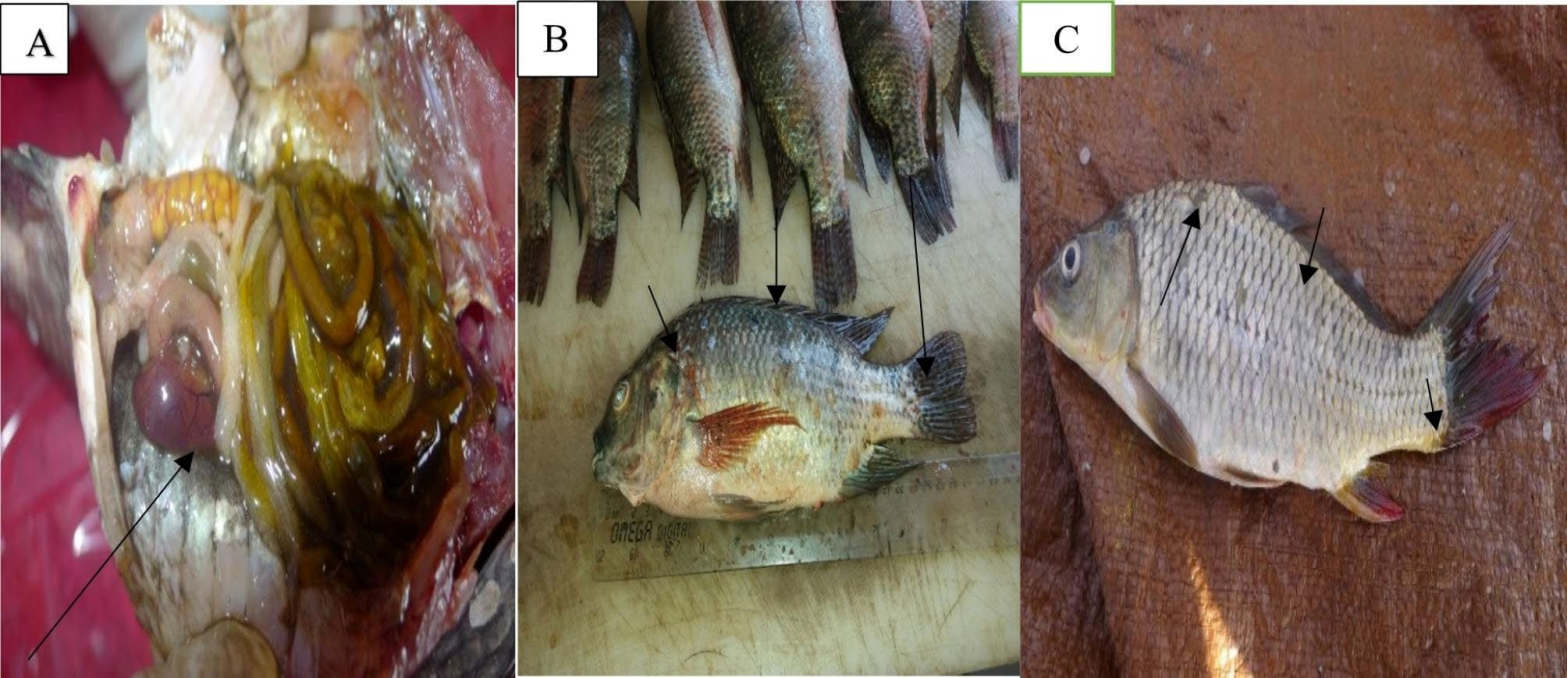
Clinical picture and post mortem findings

Fishes suspected of infection with *A. hydrophila* showed hemorrhages all over the body especially at the base of fins and tail. Clinical presentations observed include fins rot, cloudiness of both eyes, detachment of scales and skin ulceration and abdominal distention. Internally these fishes showed abdominal dropsy with reddish ascetic exudates, liver paleness and enlargement in some fishes and congested with necrotic patches in other fishes, spleen was congested, enlarged and hemorrhagic enteritis in some fishes as shown in (Fig. 1).

An arrow in (A) shows abdominal dropsy with reddish ascetic exudates (B) shows skin hemorrhage at the base of pectoral fin with hemorrhagic skin ulcer under the dorsal and tail fin and dark discoloration in the skin (C) shows skin ulcer.

### Bacteriological identification and biochemical characterization of ***A. hydrophila***

**Fig. 2:**
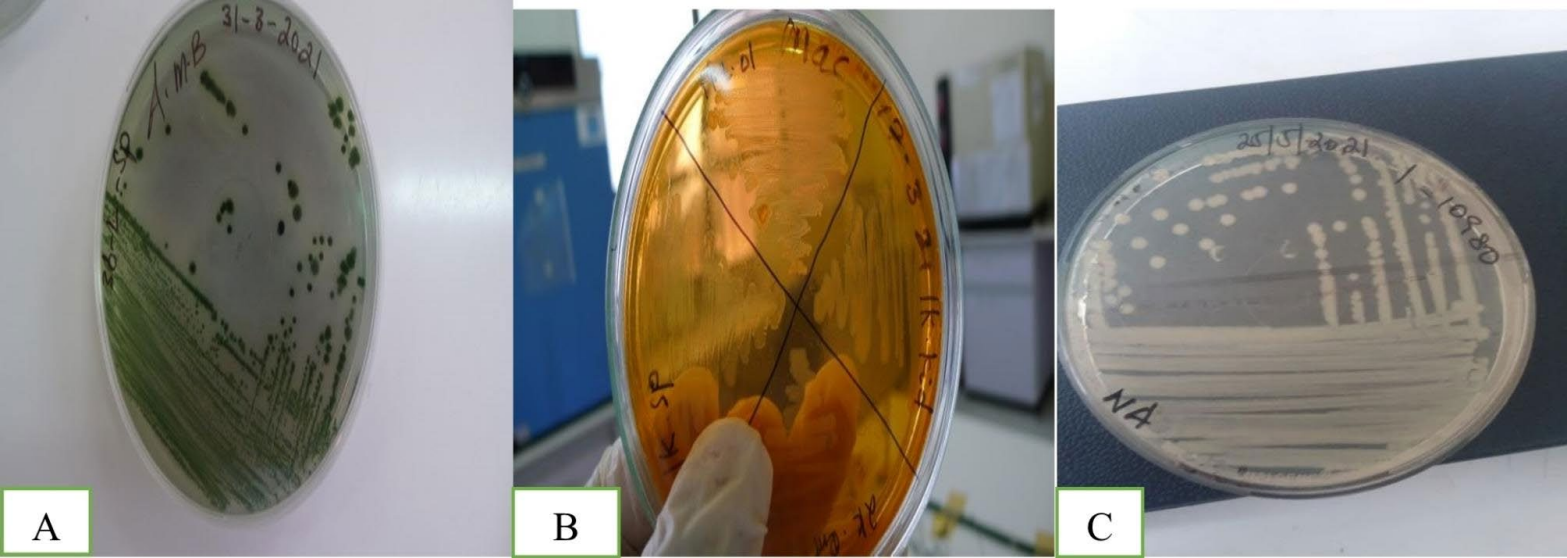
Characters of *A. hydrophila* on (**A**) Aeromonas medium base (**B**) Mac-Concey agar **C**) Nutrient Agar

The presumptive identification of the bacteria in the current study was carried out from the colony morphology over Aeromonas Medium Base, a selective medium for *A. hydrophila*. Accordingly, based on 14 morphological and biochemical tests, a total number of 81(60.45%) isolates were presumptively identified as *A. hydrophila.* They appeared rounded smooth colonies 2-3 mm in diameter and dark green with a darker center in Aeromonas medium base, pale like shaped on MacConkey agar indicated that *A.hydrophila* is unable to ferment lactose sugar and creamy white on Nutrient agar as presented in (Fig. 2). Colonies were gram-negative short rods, they gave a positive reaction for oxidase, catalase, DNase, Indole production, also ferment glucose with production of acid and gas, sugar utilization K/A, Acid production from (Sucrose and Mannitol) and Motile. They gave negative results toward xylose, urea hydrolysis, and non-lactose fermentation and produced variable results with MRVP.

### Hemolysis assay

**Fig. 3:**
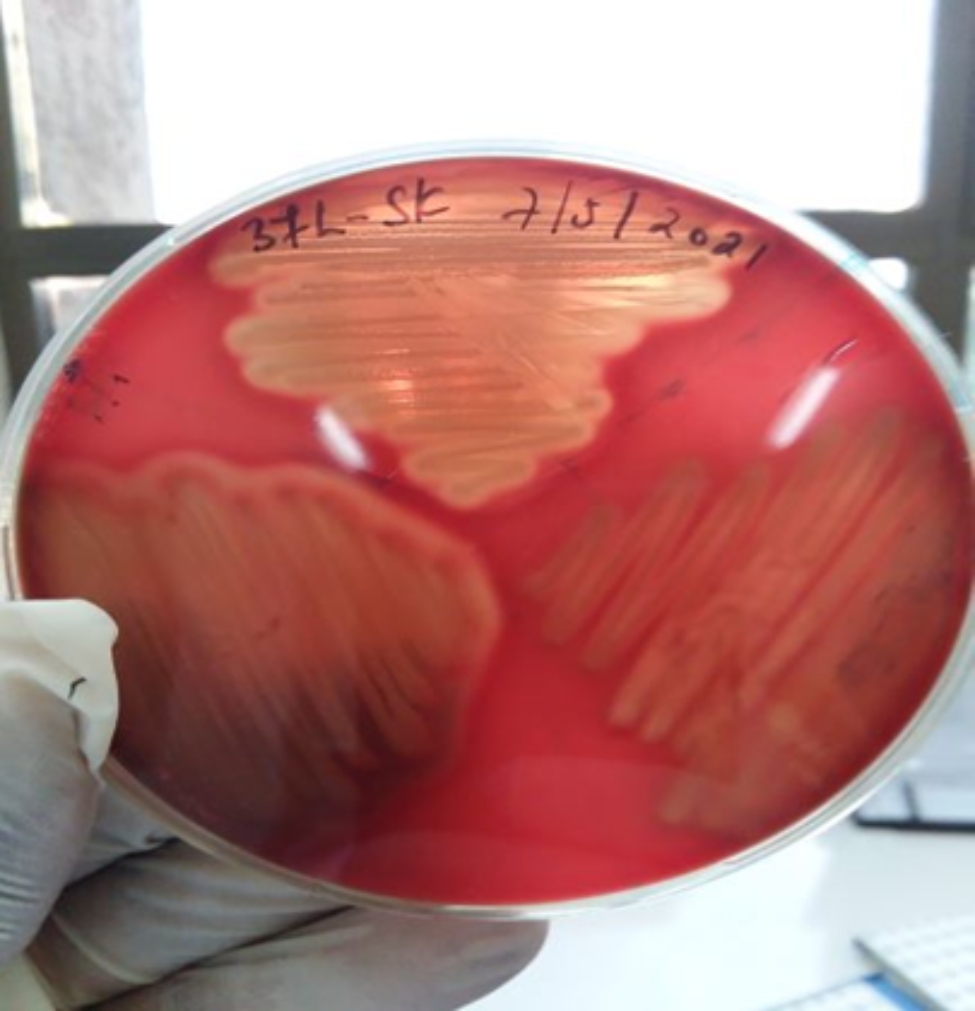
ß-hemolysis activity by *A. hydrophila* on blood agar base with 5% sheep blood

Hemolytic activity of the isolates was determined for its importance as a virulent factor. *A. hydrophila* produced hemolysis on blood agar base with 5% sheep blood. Accordingly, from the current study found that 93.33% (n = 56/60), 94.11% (n = 16/17) and 75% (n = 3/4) isolates from the life fish group, market fish and RTE fish show β hemolysis respectively and 6.66% (n = 4/60), 5.88% (n = 1/17) and 25% (n = 1/4) show α hemolysis. The hemolysis pattern results in the media displaying clear halos around bacterial colonies as shown in (Fig. 3). Hemolytic activities of *A. hydrophila* from the current study found over all isolates 92.59% (n = 75/81) show β hemolysis and only 7.4% (n = 6/81) of α hemolysis as shown in (Table 1).

**Table 1:** Hemolytic characteristics of the isolates

Hemolytic activity of *A. hydrophila* isolated from fish samples Source			
Source	Total	β	α
Live fish	60	56 (93.33)	4 (6.66)
Market fish	17	16 (94.11)	1(5.88%)
RTE	4	3 (75%)	1 (25%)
Total	81	75 (92.59%)	6 (7.4%)

β: beta, α: alpha

### Molecular detection

#### Quantitative real-time PCR detection of ***A. hydrophila*** and virulence gene

**Fig. 4:**
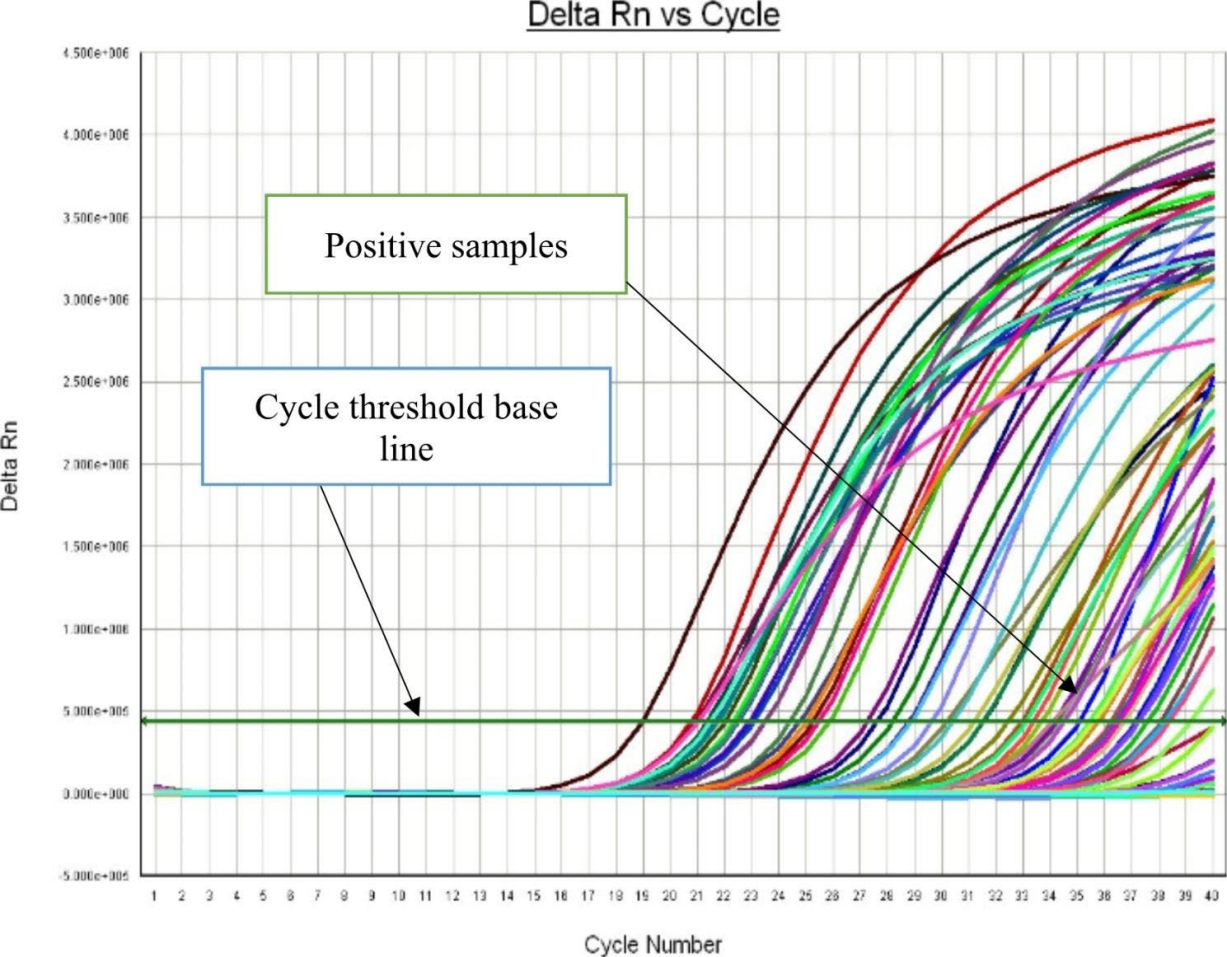
Real time PCR positive samples of *A. hydrophila*

**Fig. 5:**
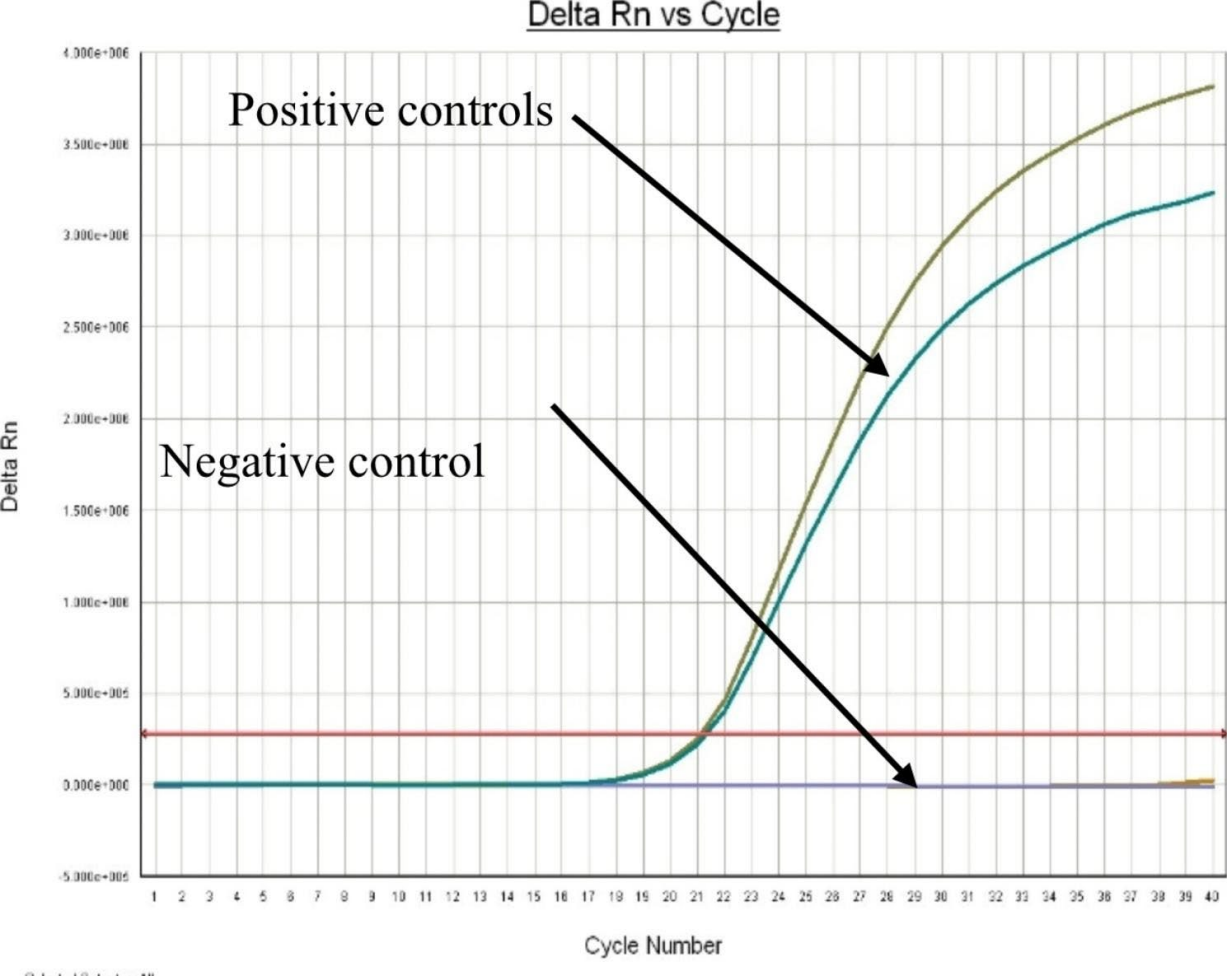
Positive and Negative Controls

**Fig. 6:**
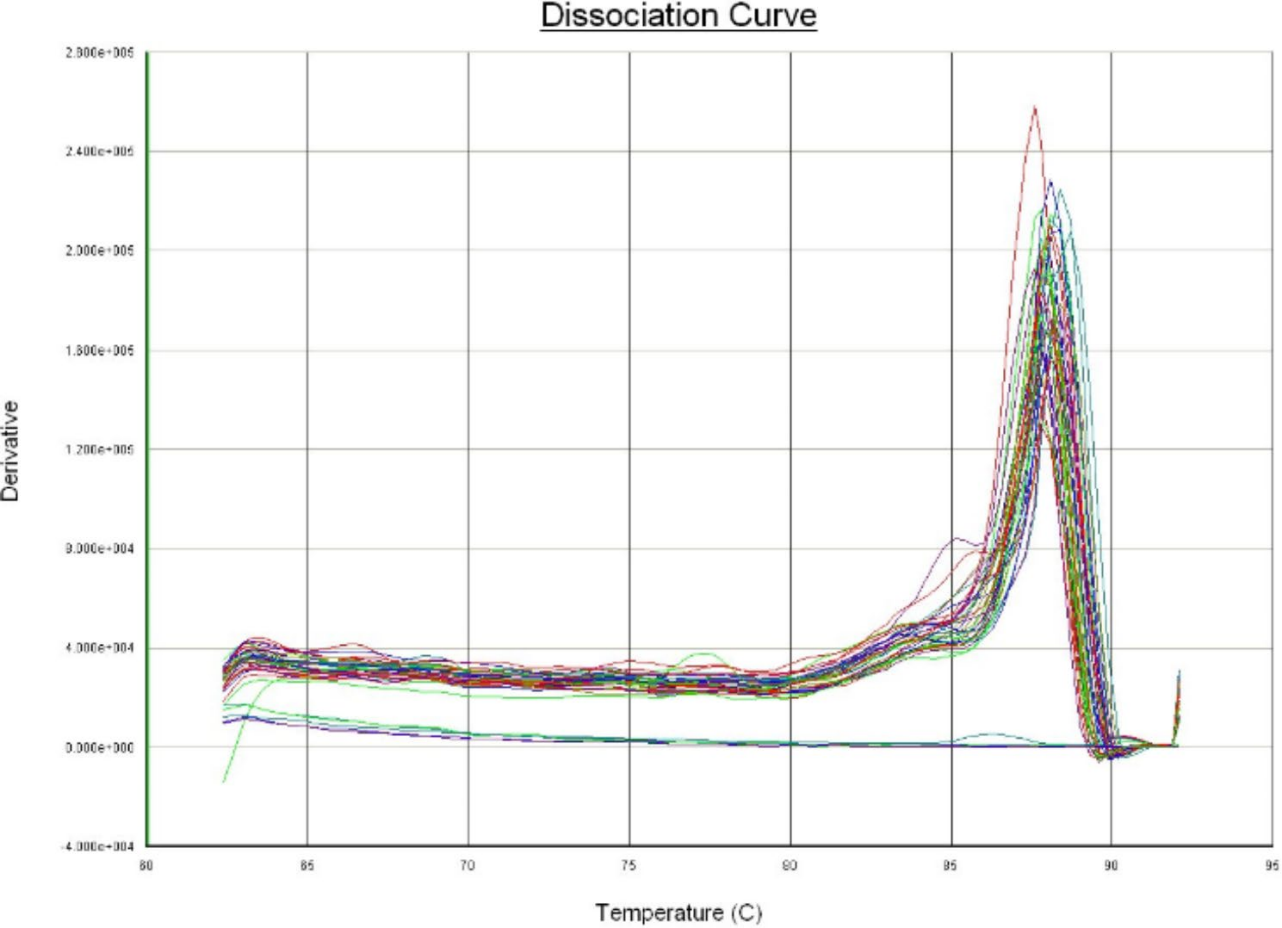
Melting curve analysis

Molecular detection with Real-Time PCR (qPCR) using specific primers based on the sequence of the ahaI gene coding for adhesive surface protein mainly present in virulent *A. hydrophila* strain. From the total of 81 *A. hydrophila* isolates, 54 were confirmed by real-time PCR for presence of the ahaI gene. The threshold cut off value for classifications of the samples as positive or negative by the real-time PCR was set to a cycle threshold (Ct) value of 34. Samples giving a Ct value of ≤ 34 with a sigmoid shape of the analysis curve were classified as positive (Fig. 4). Samples with a Ct value > 34 were classified as negative. The Ct value of real time PCR positive samples ranges between 19 and 34. A no-template control and positive control were included in every reaction (Fig. 5). The melting curve analysis of the PCR products showed typical melting profiles at 85^o^C (Fig. 6), while the negative samples did not show any melting curve.

#### Detection of ***A. hydrophila*** from different source

A total of 140 samples were collected from fish and different sources and subjected to culture on *A. hydrophila* selective media (AMB). From these, 81 (57.86%) isolates were presumptively identified as *A. hydrophila* by morphological and biochemical examination. These isolates were further confirmed as *A. hydrophila* by qPCR 54 (66.67%) positive as shown in (Fig. 4) based on specific primers on the sequence of the ahaI gene from the strain *A. hydrophila subsp. hydrophila* ATCC 7966 (Table 2).

**Table 2:** Detection of *A. hydrophila* based on source

Factors	No of sample cultured	CP	qPCR	
Live Fish	100	60	37 (61.67%)	
Market	20	17 (85%)	13 (76.47%)	
RTE	20	4 (20%)	4 (100%)	
Total	140	81 (60.45%)	54 (66.67%)	

CP: culture positive, qPCR: quantitative polymerase chain reaction

#### Detection of Aeromonas hydrophila isolated from examined fishes based on the organs

In the current study, *A. hydrophila* was detected on the basis of their organ’s location. Accordingly, the highest detection (40.54%) was assessed in both Muscle and gill, and the lowest (2.7%) was observed in Spleen (Table 3).

**Table 3:** Detection of *A. hydrophila* in respect to the organs

Organ	No of culture positive	Total qPCR positive
Muscle	17	15/37 (40.54%)
Gill	18	15/37 (40.54%)
Intestine	5	4/ (10.81%)
Spleen	8	1/37 (2.7%)
Kidney	12	2/37 (5.4%)
Total	60	37/60 (61.67%)

### Antibiogram analysis

**Fig. 7:**
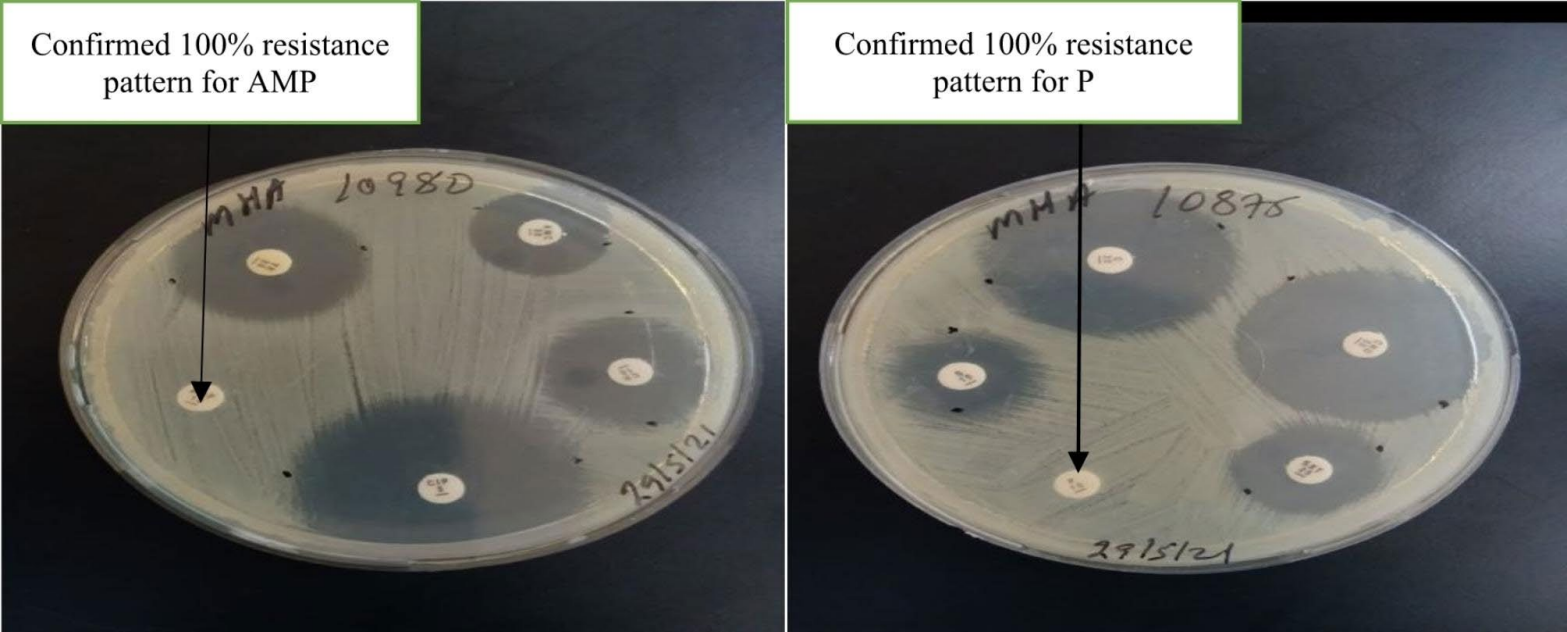
Confirmed complete drug resistance pattern for P and AMP.

In the present study, antibiogram assay for the examined *A. hydrophila* isolates concerning 10 antibiotics revealed that all the tested isolates were completely sensitive to ciprofloxacin (100%), chloramphenicol (100%) and ceftriaxone (100%). In addition, amoxicillin and penicillin did not exhibit any bactericidal activity (100% resistant) as shown in (Table 4) and (Fig. 7) and multi antibiotic resistance index of 0.18 as shown in (Table 5). The result was interpreted as sensitive, intermediate and resistant according to the National Committee for Clinical Laboratory Standards (NCCLS) recommendations for Aeromonas species [15].

**Table 4:** Antibiotic susceptibility of *A. hydrophila*

Source	Isolate ID	Antimicrobial Agents Concentration μg									
		AMP,10 μg	CN 10 μg,	AMC 30 μg	TE 30 μg	CIP 5 μg	S 10 μg	C 30 μg	SXT 25 μg	CRO 30 μg	P 10 μg
Life fish	10,881	R	S	S	S	S	I	S	I	S	R
	10,875	R	S	R	S	S	S	S	S	S	R
	10,886	R	S	I	S	S	I	S	S	S	R
	10,970	R	S	S	S	S	I	S	S	S	R
	11,060	R	S	R	S	S	I	S	I	S	R
Market fish	10,880	R	S	R	R	S	S	S	S	S	R
	10,976	R	S	R	S	S	I	S	I	S	R
RTE	10,980	R	S	R	R	S	I	S	I	S	R
	10,895	R	S	R	I	S	I	S	S	S	R
Water	11,071	R	S	S	S	S	S	S	S	S	R

**Table 5:** Frequency distribution of multidrug resistant *A. hydrophila* isolates

Resistance pattern	*A. hydrophila* isolates (no = 10)		
	**No. of** ***A. hydrophila*** **isolates**	**Percentage of** ***A. hydrophila*** **isolates**	**MAR index**
Resistance to 2	10	100	0.28
Resistance to 3	6	60	0.2
Resistance to 4	2	20	0.08
**Average MAR = 0.18**			

MAR: Multi – Antibiotic Resistance

## Discussion

Bacterial diseases are considered to be the most serious disease problem among freshwater fishes [16]. *A. hydrophila* has gained increased attention due to pathogenicity to humans and the ubiquity of the organism in the environment, food and water [17]. Isolation of *A. hydrophila* from four freshwater lake fishes along its value chain during the current study adds more evidence for the wide geographical distribution of the bacteria.

The clinical picture and postmortem findings observed in the current study of Nile tilapia were nearly similar to those described by [18–20]. The phenotypic and biochemical characteristics of *A. hydrophila* isolates recorded were in line to those reported in Bergey’s manual of determinative bacteriology [21]. Similar phenotypic and biochemical findings with current study were also reported by [3, 18, 22–24].

Hemolytic activity of the isolates was determined for its importance as a virulent factor. Accordingly, from over all isolates 92.59% show β hemolysis and only 7.4% show α hemolysis. These toxins are responsible for lethality, hemolysis and entero-toxigenicity. Their production by organisms found in food signals public health concern. The secretion of these extracellular proteins hemolysin associated with bacterial virulence (hemolytic toxins) contribute to the virulence of *A. hydrophila* in fish and human host. The bacterium could be entero-toxigenic and may be responsible for outbreaks of diarrhea if the fish are consumed without proper cooking in humans.

Molecular characterization of isolate using real-time PCR for the first time provided evidence for presence of ahal gene in *A. hydrophila* infecting fish of Ethiopia. The optimized qPCR protocol which uses ahaI gene. Accordingly, qPCR revealed presence of adhesin gene in 66.67% of the *A. hydrophila* isolated from samples. The adhesin gene is a virulence gene that code for bacterium surface protein useful to surface binding, colonization and infection of the host tissue. Targeting this adhesin gene (ahaI) constitutes an interesting and valuable study, not only to identify the specie, but also, enables future projects regarding recombinant adhesin as potential vaccine against Aeromonadaceae. From the total of 54 (66.67%) of qPCR positive samples, 37 (68.51%), 4 (7.4%), and 13 (24.07%) were from Fish source, RTE, and market fish respectively with no disease outbreak reported in all lakes at the point in time. As it was explained by Gilda [25], that disease occurrence in fish is a function of the pathogen, host and the environment. These results were at par with those reported by [26] in Iraq who found that over all detection rate of 65% *A. hydrophila* [27], in Berlin, Germany who found 63.% cytotoxin producing *A. hydrophila*. However, lower prevalence were detected by [28] in Tamilnadu, India who found 40% of detection rate; [29] who found 40% *of A. hydrophila* from wild fish in Assiut, Egypt [23], in Moshtohor Egypt, who detected the total prevalence of bacterial infection (55.3%) [30], who found the prevalence of *A. hydrophila* 47% in Alexandria, Egypt, and [31] in Brazil who found the total prevalence of 46.66% *A. hydrophila*. However, a higher prevalence of *A. hydrophila* (95.06%) was reported by [32] in LiebefeM-Bern [33], in Kafrelsheikh governorate, Egypt who found a total prevalence of 75%. Variations in the incidence level of *A. hydrophila* in the fish worldwide can be attributed to sampling time and geographical range [34]. Difference in the current study may be attributed to the number of examined fish, the size of fish and environmental conditions, geographical range, seasons of the study, sensitivity, and specificity of the techniques used to identify the bacteria.

Overall *A. hydrophila* (24.07%) contaminations in the market fish and RTE (7.4%) was observed in the current study. These results are in accordance with [35], who identified *A. hydrophila* (22.6%) from market fish in Ankara (Turkey). In Brazil [36] who detected 22.9% *A. hydrophila* from market fish samples. However, lower prevalence was detected by [37] Santos et al. (2002) who isolated 13% *A. hydrophila* from market fish samples in Brazil. Different studies have reported inconsistent detection rates of *A. hydrophila* for instance, Minana identified 2% of market fish in Spain, While, in India, 15.6% detection rate of *A. hydrophila* was reported in marketed fish samples by [38]. However, a higher prevalence recorded by [39] in Riyadh, Saudi Arabia who found 34% from fish market samples [29], who found (40%) of *A. hydrophila* from market fish in Assiut, Egypt and Attia [40], who reported overall higher *A. hydrophila* (51.4%) contaminations in the market fish in Sharkia Governorate, Egypt. This may be due to post-harvest contamination during selling through fishermen improper handling and transportation from the catching area. Fish in retail in the current study area are considered potential source for infection of human consumers. Although, the source of the organism may be ambient environment, secondary contamination during catching, handling and transportation may also contribute for its distribution.

Fish products (“leb-leb”, fish salads, “gulash”, smoked fish, etc.) are some of the most popular RTE choices in Ethiopia. Concerning the detection of *A. hydrophila* in RTE fish, the current study revealed 7.4%. This results are in accordance with [40], who detected the prevalence of *A. hydrophila* in RTE grilled fish 8.6%. Mohamed [41], in Assiut Egypt, reported that *A. hydrophila* 20 and 10%, detection rate in grilled and fried fish samples respectively. A lower percentage (2.3%) of *A. hydrophila* was reported in RTE fish product in India by Gupta. Whereas, a higher percentage (77.3%) in RTE fried fish in India was also reported by [42]. The contamination rate in RTE fish may suggesting contamination after cooking caused by lack of hygiene, contaminated water or contaminants from uncooked produce. The presence of *A. hydrophila* in RTE products again may be attributed to rapid grilling which could be insufficient to kill *A. hydrophila* that may be present in raw fish before preparation.

Regarding the frequency of detecting *A. hydrophila* from the different parts of the fish, out of 37 (68.51%) fish tissue samples, it was noticed that the highest (40.54%) gene detection was recorded from both gill and muscle respectively, (10.81%) from intestine, 5.4% from kidney and the lowest (2.7%) gene detection was recorded from spleen. The high proportion of infection in gills and muscle in comparison to other organs is due to the exposed nature of the organ to microbiota. The current findings are supported by the observations of [32, 43–45] who reported that *A. hydrophila* has detected from wild fish, pond cultured edible and ornamental fish from different parts of the fish. These attributed to the ubiquitous nature of the microorganism in the aquatic environment. The predominance of *A. hydrophila* in the gill and muscle of fishes may be attributed to the presence of *A. Hydrophila* in contaminated water in which the fish lives [5].

With the steady expansion of the fishery industry, the vast use of antibiotics will be unavoidable. The continuous and extensive use of antibiotics in humans also led to the emergence of antimicrobial-resistant strains worldwide [46]. Ten antibiotics namely; Ampicillin, Penicillin, Tetracycline, Ciprofloxacin, Chloramphenicol, Streptomycin, Gentamicin, Ceftriaxone, Amoxicillin-clavulanate, and Trimethoprim-Sulphamethoxazole were used in the current study mainly due to their routine usage in veterinary and human medicine. Fish treatments are not practiced almost in all fishery and aquaculture sectors of Ethiopia but, Tetracycline is commonly applied for the treatment of bacteremia in fishery research centers of Ethiopia (observation).

In the present study, antibiogram assay for the examined *A. hydrophila* isolates concerning 10 antibiotics revealed that all the tested isolates were completely sensitive to ciprofloxacin (100%), chloramphenicol (100%) and ceftriaxone (100%). In addition, amoxicillin and penicillin did not exhibit any bactericidal activity (100% resistant) against the tested isolates. These results are nearly agreed with those obtained by [10, 11, 16, 19, 30, 47–50]. Freshwater streams are usually receptors of many industrial, domestic and agricultural wastes, which could contain antimicrobial agents and antimicrobial-resistant bacteria [51, 52]. Due to diverse microbial population in such ecosystems freshwater environment provides favorable conditions for the spread of antimicrobial resistance. The resistance to penicillin in *A. hydrophila* mainly attributed to β -lactamase production that encoded in their chromosomes. The antibiotic resistance has a public health concern it mainly results from the improper intensive use of antibiotics [10]. The aeromonads have been regarded as being universally resistant to penicillin [10], in the current study penicillin and ampicillin resistance were confirmed. In the present study the multi-drug resistant (MAR) of the *A. hydrophila* were 0.18 and this finding are in accordance to the previous study of [10, 11].

## Conclusions

The present study provided first evidence infections of fish and fish products with virulent *A. hydrophila* strains. The pathogen was isolated and identified in 81 samples. On phenotypical assessments 92.59% (n = 75) of the isolate expressed virulence trait of ß – hemolysis. Molecular characterization using real-time PCR revealed presence of the adhesin gene (ahaI) in 54 (66.67%) of the isolates. Meanwhile, antimicrobial susceptibility test on selected *A. hydrophila* strains revealed the presence of resistance to amoxicillin and penicillin. The phenotypic and genotypic analysis provided epidemiological evidences for dissemination of a virulent *A. hydrophilia* strain among the fish population in rift valley lakes. The detection of the pathogen in hemopoetic organ of the sampled fish population is alarming for potential outbreaks. The identified *A. hydrophilia* isolates carry virulence trait that aids in colonization, infection and pathogenicity with ability to resist antibiotics commonly used in human and veterinary medicine. *A hydrophila* is a zoonotic emerging pathogen and fish in lakes and fish products from Lake Koka, Zeway, langano and Hawassa are a potential sources of infection for humans in the area.

## Methods

### Study area

The current study was conducted in selected Rift Valley Lakes of Ethiopia, Koka, Ziway, Langano, and Hawassa from November 2020 to June 2021 from lake fishes, market fish and Restaurants of respective areas.

**Figure:**
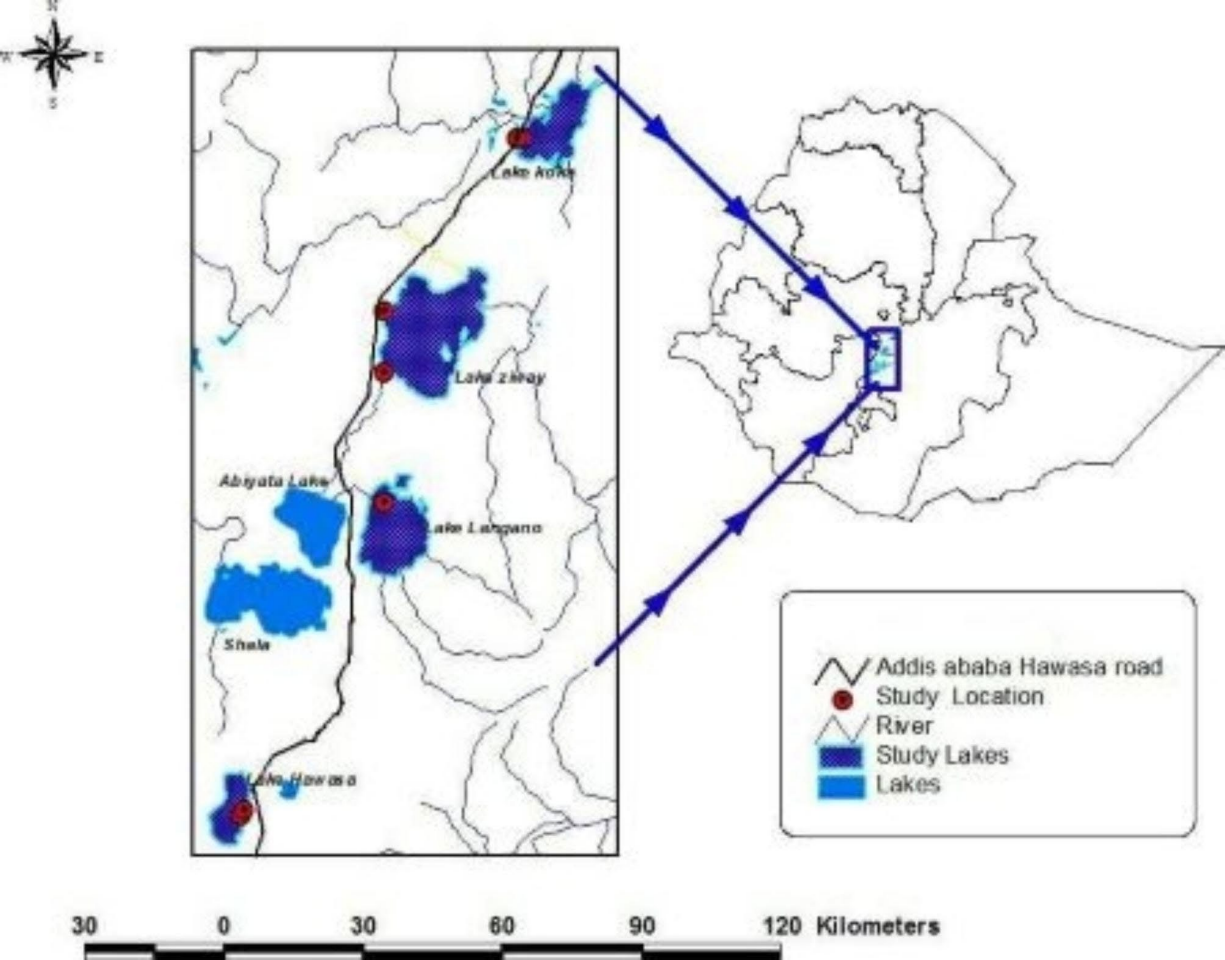
Map of the study area

### Study population

The present study was conducted on the Nile tilapia (*Oreochromis niloticus*), fish host biometric data including (average total body length TL, 246 mm, 5 mm accuracy) and weighed (average total body weight of 282.3 g) collected from the lakes, different fish markets and restaurants at study areas. Nile Tilapia were selected because of the fish population density and the trends of consumption preferences in the area [1].

### Study design

A total of 140 fish samples were collected from different sources with a cross-sectional study from November 2020 to May 2021 at Koka, Ziway, Langano and Hawassa Lakes. The lakes were selected because of the bulk of the fish catch that contributes to 79% of the total fish catch in the country [1]. Restaurants in respective areas based on the accessibility to public transport transit areas and presence of recreational activities around the lake.

### Sampling procedure

Purposive sampling strategy was followed in selecting fishes i.e. fish with suggestive lesions (hemorrhages on the external surface, the base of pectoral and tail fin, ulcer on the skin, abdominal distention, unilateral or bilateral exophthalmia, prolapsed anus, and fin rot) of *A. hydrophila* infection were picked for sampling. As per mentioned somewhere tissue samples (muscle, gill, intestine, spleen and kidney) were collected from those fish having suggestive lesions [18–20]. All the fishes were caught using gillnets with mesh size ranging from (10 to 14 cm) that were used for the exploratory fishing work at the lakes. Samples were carried in Autoclavable sterile plastic bag containing water from the lake where they were caught and transported alive to Batu fishery and other aquatic life research center laboratory and Hawassa University Biology Department laboratory for post mortem examination and were analyzed immediately.

### Clinical examination

Sampled fish were subjected to the clinical examination of the gross external signs as described by [18, 53, 54]. Fish was killed by transecting the spinal cord behind the skull. Autopsy and examination of the internal organs were carried out according to the method described by [55]. The organs sampled was muscle, gill, intestine, kidney, and spleen for bacterial culture and molecular analysis. First, the external body surface of the fish was examined for the presence of lesions, the gills, tail, and fins was observed for visible signs of infection and samples from muscle and gill were taken aseptically. After opening the body, the internal organs were exposed with care not to puncture any part of the intestinal tract by using ventral approach. In the absence of any visible lesions samples of a kidney, spleen and Intestine was taken after searing the surface of the organs with a hot scalpel blade. 2gm of each specimen were aseptically taken into the falcon tube (50ml) containing 20ml of alkaline peptone water PH 8.5 (Oxoid, England) which were kept cool at 4^o^c.

All raw fish was purchased and collected from traditional markets and supermarkets from respective towns by purposive selection based on the amount of fish stock kept on specific seller, market availability and customer’s choice store based on informal collection of data in the area. Each market fish sample was individually packed in a clear sterile polyethylene bag immediate after sampling while, RTE fish samples collected as take away order and bagged in sterile plastic bags. 2gm of each specimen from market fish and RTE added to falcon tube containing 20ml of peptone water and preserved in an icebox. All the specimens from fish, market and RTE were labeled and transferred to the laboratory under aseptic condition with a minimum of delay in Batu and Hawassa University laboratories and finally brought to National Animal Health Diagnostic and Investigation Center (NAHDIC) with Electrical cooler jugs (icebox) for further studies.

### Bacteriological examination (phenotypic identification)

A standard operating protocol was used for isolation and identification of *A. hydrophila* from fish and water samples [30, 56]. Aseptically taken 2gm of each fish sample (muscle, gill, intestine, kidney, and spleen) was thoroughly mixed (vortexed) from 20ml of samples in alkaline peptone water which is used as enrichment and transport media as per the method described by [22]. The homogenates were incubated for 24 h at 35 °C [53]. A loop-full from each enriched homogenate was streaked on to Aeromonas Medium Base (Oxoid, England), for each 500 ml Aeromonas medium base, 1 vial of ampicillin selective supplement was used and incubated for 24 h at 35 °C, a single colony from each suspected isolate was picked up and re-streaked on a new plate of its perused selective culture media and re-incubated at the same conditions. Presumptive colony from Aeromonas medium base inoculated in to Brain heart infusion broth (Oxoid, England) and incubated for 18-24 h at 35^o^C, then loop-full from the broth cultured on Nutrient agar media and incubated for 24 h at 35^o^C, each pure colony from the nutrient agar medium used as a stock culture for further biochemical identification [57].

*A. hydrophila* were identified biochemically to species level based on colonial characteristics (colony morphology and arrangement) and by using 14 chosen biochemical test including gram staining of the microorganisms, cytochrome oxidase, catalase, motility, sugar utilization, indole, methyl red test, hemolysis production, Voges- Proskauer test, DNase test, gas production from Glucose, acid production from Sucrose, Mannitol and Xylose. Then the phenotypic and biochemical characteristics of the isolates were characterized according to the guideline indicated in Bergey’s manual on fish and other aquatic animal practical identification manual [22].

### Phenotypic characterization of ***A. hydrophila*** virulence determinants

The collected isolates were examined for their hemolytic activity on 5% whole sheep blood agar medium and results was recorded after 24 h of incubation at 35 °C and checked for the type (α or ß) of hemolytic activity.

**Molecular Detection of *A. hydrophila***.

#### DNA extraction

Genomic DNA was extracted using the DNA extraction kit (DNeasy kit, Qiagen, Germany) following the manufacturer’s instructions. Qiagen DNeasy DNA extraction protocol for bacterial cultures adapted from Qiagen DNeasy handbook, 2020. Briefly, 200 μl of the sample suspension was incubated at 70 °C for 10 min after the addition of 20 μl of proteinase K and 200 μl (AL) Buffer or lysis buffer by vortexing. Then, 200 μl of 100% ethanol was added to the lysate and mixed thoroughly by vortexing. Washing and centrifugation of the sample was performed following the manufacturer’s recommendations. Then, nucleic acid was eluted with 200 μl of elution buffer provided in the kit.

#### Real-time qPCR amplification

Real-time qPCR was performed using a thermo cycler for real-time PCR (Applied Bio systems - Model Real time − 7500) and the marker used was Eva green Super mix (Bio-Rad, USA). The Amplification reactions were performed in a reaction mixture of 20 μl volumes consisting of 1 μl of each ahaI primer (F and R), 10 μl of 10x master mix including buffer, MgCl2, dNTPs, Evagreen and DNA polymerase, 6μL of RNase-free distilled water and 2 μl of genomic DNA template. The PCR program consisted of an initial step at 50 °C for 2 min and 95 °C for 10 min, followed by 40 cycles of denaturation at 95 °C for 15s and annealing at 60 °C for 1 min. At the end of each cycle, a DNA melting curve of the amplified products was performed between 65 and 95 °C, 95^o^C for 15 s, 65^O^c for 1 min and 95^o^C for 15 s with an increase of 0.5 °C in a stepwise manner to evaluate the melting temperature (Tm) and to check the random amplification of untargeted regions.

### Primer design

Gene specific primers used here were previously described by [58] based on the sequence of the ahaI gene from the strain *A. hydrophila subsp. hydrophila* ATCC 7966. Sequences are shown in Table 6.

**Table 6:** Primer sequences (5’ to 3’) used to amplify the gene ahaI in *A. hydrophila*, yielding a 200 bp amplicon

Primer	Primers sequences (5’-3’)	T_m_ (°C)	Reference
ahaI Forward	5- GAGAAGGTGACCACCAAGAACA-3	57.8	(58)
ahaI Reverse	5- GAGATGTCAGCCTTGTAGAGCT-3	54.2	

### Antibiogram analysis

*A. hydrophila* strains was subjected to antibiotic sensitivity test using the Kirby-Bauer disc diffusion method according to the National Committee for Clinical Laboratory Standards (NCCLS) recommendations for Aeromonas species [15]. *A. hydrophila* isolates was inoculated in TSB and incubated at 35ºC for 16-20 h, the turbid broth was inoculated in Muller Hinton broth (Oxoid, CM0405), the turbidity was adjusted according to McFarland obesity tube No. 0.5. Isolates was streaked on Muller Hinton agar (Oxoid, CM0337) and disks were placed, incubation was done at 37ºC overnight. The used antibiotics were Amoxicillin-clavulanate (AMC, 30 μg), penicillin (P, 10 μg), Ampicillin (AMP, 10 μg), Ceftriaxone (CRO, 30 μg), Gentamicin (CN, 10 μg), Streptomycin (S, 10 μg), Tetracycline (TE, 30 μg), Ciprofloxacin (CIP, 5 μg), Trimethoprim-Sulfamethoxazole (SXT, 25 μg) and Chloramphenicol (C, 30 μg). Antimicrobials are selected based on the importance and common use in preventing and treating diseases in both veterinary and human medicines. After a period of 24 h. incubation, the zones of inhibition were compared and measured according to the manufacturer’s instruction [15]. The result was interpreted as sensitive, intermediate and resistant according to the reference values.

The formula below is used to calculate the Multiple Antibiotic Resistances (MAR index) of the present isolates against tested antibiotics.

MAR index = X/(Y×Z).

Where; X–Total of antibiotic resistance case.

Y–Total of antibiotic used in the study.

Z–Total of isolates. When the use of antibiotics is seldom or of low dose use for animal treatment, the MAR value is usually equal to or less than 0.2. In contrast, the elevated rate of use or the high risk of exposure of antibiotics for animal treatment will yield an MAR index value which is more than 0.2.

### Data management and analysis

The collected data were entered into Excel spreadsheet (Microsoft® office excel 2016) spread sheets and descriptive statistics was used.

## Data Availability

The datasets used and analyzed during the current study are available from the corresponding author on reasonable request.
